# Invasion dynamics of Asian hornet, *Vespa velutina* (Hymenoptera: Vespidae): a case study of a commune in south-west France

**DOI:** 10.1007/s13355-016-0470-z

**Published:** 2017-03-17

**Authors:** Daniel N. Franklin, Mike A. Brown, Samik Datta, Andrew G. S. Cuthbertson, Giles E. Budge, Matt J. Keeling

**Affiliations:** 1 ZEEMAN Institute: SBIDER, University of Warwick, Coventry, CV4 7AL UK; 2 School of Life Sciences, University of Warwick, Coventry, CV4 7AL UK; 3 National Bee Unit, Animal and Plant Health Agency, Sand Hutton, York, YO41 1LZ UK; 4 Mathematics Institute, University of Warwick, Coventry, CV4 7AL UK; 5 Fera, Sand Hutton, York, YO41 1LZ UK

**Keywords:** Asian hornet, Honeybee, Pest, Invasion, Inferred dynamics, Detection, Control

## Abstract

Asian hornet, *Vespa velutina* Lepeletier nests were discovered in 2007 in Andernos-les-Bains on the south-west coast of France, 3 years after the first reported sightings in France. The number of nests increased in the commune over the following 7 years, despite local authorities enacting a destruction policy. The nests existed in close proximity to one another leading to a high density of over 10 nests per square kilometre in urban areas. New information on the chosen habitat for nests is presented, and the differences between primary and secondary locations are evident, with primary nests mostly occupying buildings and man-made structures, while secondary nests were found on trees. Using Bayesian inference methods, we fit a basic model to the observational data, which allows us to estimate key demographic parameters. This model fit is highly informative for predicting *V. velutina* spread and colonisation of other at-risk regions, and suggests that local control has a limited impact on the spread of *V. velutina* once established within a region.

## Introduction

Insects are vital to agriculture globally through their role as pollinators of crops. It has been estimated that the economic value of pollinators worldwide is 153 € billion (Gallai et al. 2009). The European honeybee, *Apis mellifera* Linnaeus is the most widely managed pollinator in the world, with recent estimates suggesting this single species may contribute nearly half of global crop pollination services (Kleijn et al. 2015).

Recent changes in the population sizes of honeybees and other pollinators are difficult to quantify. There is clear evidence for severe regional declines in domestic honeybee stocks in the USA (Council 2007; vanEngelsdorp et al. 2008) and Europe (Potts et al. 2010). The number of honeybee hives worldwide has increased by 45% in the last half century, yet the demand for pollinators to drive pollinator-dependent agriculture has increased by 300% in the same time period (Aizen and Harder 2009). This increase is due to economic and political factors, such as increasing populations and demand for food, rather than biological reasons. Honeybees have always faced biological threats; however, the need for them in their role as pollinator is now more pertinent than ever, and so the need to reduce the biological threats they face has become critical. The increase in managed honeybee hives has taken on extra importance due to the decline in wild and feral populations (Kraus and Page 1995; Moritz et al. 2007), and of other wild bee species (Goulson et al. 2008; Williams and Osborne 2009).

A recent threat to honeybees in European countries is the Asian hornet *Vespa velutina* Lepeletier, (also known as the yellow-legged hornet), which preys primarily upon honeybees. Where it has been measured, bees contribute two thirds of *V. velutina’s* diet in an urban environment (Villemant et al. 2011). *Vespa velutina* is the first Vespidae predator accidentally introduced from Asia into Europe (Rortais et al. 2010; Roy et al. 2011), and was first observed in 2004 in south-west France (Rortais et al. 2010). Since then *V. velutina* has spread rapidly, and was reported in Spain in 2010 (López et al. 2011), Portugal in 2011 (Grosso-Silva and Maia 2012) and Italy in 2013 (CABI 2013). *Vespa velutina* was also detected in Belgium in 2011, but was not reported in 2012 (Rome et al. 2013). In 2014 a single nest was identified in Germany (Orlov 2014). The native range of *V. velutina* is Asia, from north-eastern India throughout southern and central China as far as Taiwan and as far south as Indonesia (Archer 1994). It was introduced into South Korea in 2003 where it has become an invasive pest (Choi et al. 2011; Jung et al. 2008), it also invaded Japan in 2012 (Ueno 2014). The most recent incursion has been into Great Britain in the summer of 2016 (National Bee Unit 2016), with a single nest located and destroyed followed by the sighting of another individual hornet likely to belong to another colony. It has been shown that an invasion can be initiated by very few or even a single mated female hornet (Arca et al. 2015).

In Asia, *V. velutina* has been reported to limit colony development of European honeybees by the persistent predation of adult bees (Shah and Shah 1991). In China, a study monitoring the predation of the Asian honeybee *Apis cerana* Fabricus and the European honeybee, *Apis mellifera*, showed that *V. velutina* were 3 times more likely to predate upon *A. mellifera* colonies over *A. cerana* colonies, and the final hawking success rates were approximately 3 times higher for *A. mellifera* foragers than for *A. cerana* (Tan et al. 2007). The observed predation is relatively continuous.

The managed and domesticated honeybee in France and the rest of Europe is *A. mellifera*. This lacks the defensive abilities of *A. cerana* such as heat-balling, (Ken et al. 2005; Ono and Sasaki 1987) and wing shimmering (Koeniger et al. 1996), nor does it possess defensive behaviours such as increased guard bees and changed flying behaviour (including reduced foraging when *V. velutina* is present) (Tan et al. 2007). This is in direct contrast to *A. cerana*, which has co-evolved with *V. velutina*. Indeed, attacks by *V. velutina* can lead to death of *A. mellifera* colonies (Ken et al. 2005). A recent study suggests that *V. velutina* may be more inclined to prey upon *A. mellifera* colonies with the lowest demonstrated defensive behaviours (Monceau et al. 2014a).

*Vespa velutina* nests are founded by a single mated queen that has overwintered, known as a foundress. At the end of the summer, each successful nest will produce multiple foundresses, which are mated and hibernate over winter while the rest of the colony dies. *Vespa velutina* will hibernate seemingly anywhere that is small and dark. Due to this they can be transported long distances while hibernating; this is how *V. velutina* is thought to have been introduced to south-west France, via a shipment of Bonsai pots from China (Villemant et al. 2006), and likely how they were then introduced into Belgium, Portugal and Italy, due to the distances from the source nests in France. The following spring the foundresses emerge, disperse over a range of distances and form new primary nests; although the precise behaviour is not well understood. This early dispersal is thought to be the primary route by which a wave of invading *V. velutina* moves through the local landscape.

**Fig. 1:**
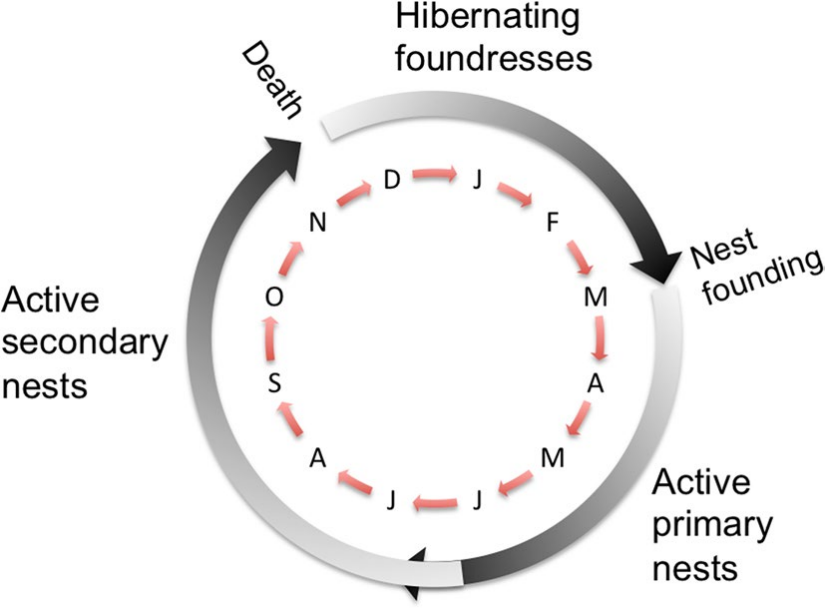
Basic life cycle of *Vespa velutina*. Primary nests detected after the end of July were considered inactive. Secondary nests detected after the middle of November were considered inactive

Primary nests are nests that the founding queen builds to lay the initial eggs: these can be considered temporary nests until the first workers emerge. As the colony grows, a larger nest is required, and the growing colony will abandon the primary nest to build a new, much larger, secondary permanent nest. This happens by the end of July (Monceau et al. 2014b). As such, a single colony will inhabit two nests, one after the other. In this paper, a nest will refer to the physical structure in which a colony lives. The secondary nest is inhabited until the males and workers die in late autumn, and the foundresses go into hibernation. Hibernation does not take place in the secondary nest. The foundresses disperse and find their own suitable hibernation location. Once this has happened the secondary nest is then empty and inactive. Figure 1 provides a graphical representation of the life cycle of *V. velutina*.

The only existing literature on the spread of *V. velutina* in France reported that 49% of discovered nests were in urban or periurban areas, 43% of nests were in agricultural areas, 7% in forests, and 1% in areas that could be classified as wetlands (Villemant et al. 2011). The previous literature of nest habitats in France reported that approximately 90% of nests were on trees, around 10% were in buildings and less than 1% were underground (Rortais et al. 2010).

Despite data collection across France since the invasion of *V. velutina* began there is little literature on the spatial spread or growth in local population, with limited quantitative analysis. Monceau and Thiéry (2016) investigated the spacing between nests from the same data, finding little of note. Here we characterise the spatial spread, population growth and carrying capacity within Andernos-les-Bains, a commune inside the invaded area. The present paper should be useful for predicting rates of spread and density in other territories at risk or in the early stages of invasion such as Great Britain.

## Materials and methods

We analysed data on the number and location of *V. velutina* nests in Andernos-les-Bains, on the south-west coast of France, from 2007 to 2014 inclusive. Nest detection combined reporting of nests by members of the public, beekeepers and the local authority. The inconsistency in searches across time implies a degree of inherent under-reporting, as with many epidemiological datasets. There was no formal searching for nests. From 2007 to 2009 and again in 2014, the structure that the nest was discovered in was recorded. From 2009 onwards, whether discovered nests were primary or secondary was recorded.

The collectors of the data identified a nest as primary or secondary based on its size; however, nest size was not available to the authors. The timing of the *V. velutina* life cycle allowed us to infer the nature of the discovered nests that were not recorded as either active or inactive (only the 2007 nests were recorded with such a classification). Nests identified as primary nests discovered after the end of July were assumed abandoned (inactive), therefore the destruction of the nest did not represent the destruction of a colony. Secondary nests (or nests without a primary/secondary designation) destroyed after the middle of November were also considered inactive (Monceau et al. 2014b), and did not contribute to the destruction of a colony (Fig. 1). Inactive secondary nests were assumed to have already produced hibernating foundresses (hibernating elsewhere), which would contribute to the number of nests in the following year.

To determine the total number of reproductive colonies in the commune in a given year, the number of colonies was calculated as the number of primary nests that were destroyed when active, plus the total number of secondary nests found (either active or inactive). Only the primary nests that were destroyed when active were included, as those that were destroyed when inactive were assumed to have already created a secondary nest. As the data did not specify whether nests in 2007–2008 were primary or secondary, the number of nests in these years was taken as the number of detected colonies.

### Terrain of Andernos-les-Bains

The terrain of Andernos-les-Bains was obtained from the EU Corine Land Cover data (EEA 2014). Approximately 48% of this terrain is considered urban land of some form, 29% is forest and 22% is woodland scrub found mostly in the east of the commune. To the south-west of the commune is the sea, while there are other urban environments to both the north-west and south-east. The area of the commune is 20.59 km^2^.

### Parameter inference

In order to gain a greater qualitative understanding of the results we developed a simple model that captures the life cycle of *V. velutina* and can be matched to the available data on the number and type of detected nests. The model describes both the absolute number of colonies generated each year, as well as the detection (and destruction) of primary and secondary nests.

We assumed that the number of colonies and hence primary nests in a given year (*P*_*y*_) is Poisson distributed with a mean that is related to the number of successful secondary nests the year before (*S*_*y−*1_) incorporating a density dependence:

$$P_{y} = {\text{Poisson}}\left( {\frac{{rS_{y - 1} }}{{1 + S_{y - 1} /\kappa }}} \right)$$

We then assume that active primary nests, active secondary nests and inactive secondary nests are all discovered with given probabilities that are allowed to vary linearly over the period of observation. Only secondary nests that are not discovered (and destroyed) when active are considered successful. This leads to an inference problem involving the two demographic parameters (*r* and *κ*) the intrinsic growth rate and carrying capacity, parameters that capture detection of different nest types, and the total number of primary nests at the start of each year. We utilised a Metropolis–Hastings MCMC (Markov chain Monte Carlo) method to infer these parameters from the data in a Bayesian framework (Chib and Greenberg 1995; Gilks 2005). Due to the interaction between detection, destruction and the invasion dynamics, simpler methods of calculating a population carrying capacity are not suitable.

## Results

**Fig. 2:**
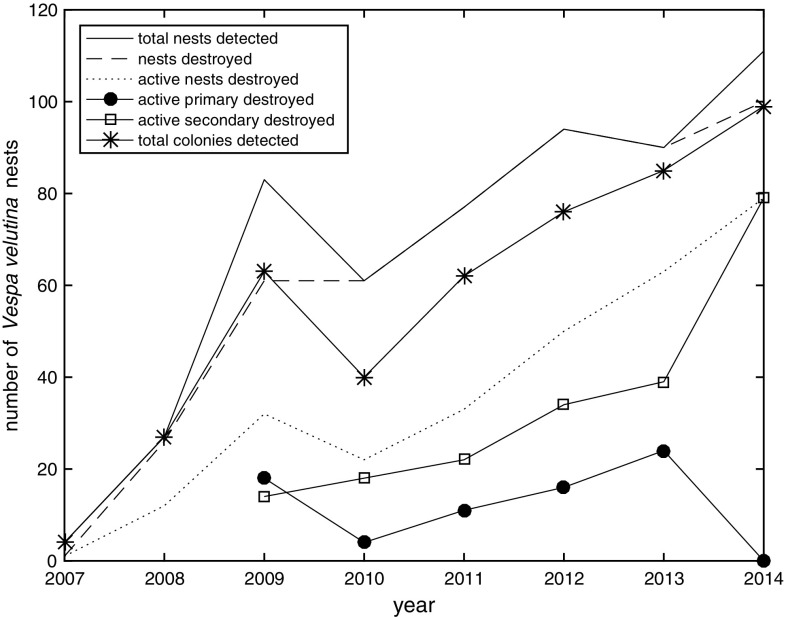
Yearly totals of *Vespa velutina* nests found and destroyed over the period 2007–2014

The initial discovery was of four *V. velutina* nests in 2007, three of these were discovered on oak trees, and one on a pine tree (Table 1). The first was discovered on 6th October 2007, but was already inactive. Given the discovery likely led to efforts to locate other nests, only one was found soon after (22nd October 2007), which was active and was destroyed; however, the other two nests were not found until the winter, suggesting that at least three nests successfully produced foundresses for the following year. The number of nests discovered increased dramatically over the next 2 years (Fig. 2) to 27 in 2008 and 83 in 2009, suggesting a high reproductive ratio between years. Although 26 of the 27 nests discovered in 2008 were destroyed, only 12 of these were destroyed at a time they were likely to be active (making the assumption that all were secondary) meaning the remaining 15 likely produced hibernating foundresses that began new colonies. The total number of discovered nests fell to 61 in 2010; this could be attributed to the relatively large number of active nests that were destroyed in 2009, although multiple other factors such as climate or sampling effort could also have played a role. From 2010 there was a trend of increasing numbers of nests being discovered each year; this could either have been due to increasing number of *V. velutina* colonies or increasing efficiency in detecting nests, or a combination of the two. The number of detected colonies followed the same pattern as the number of nests apart from in 2013 (Fig. 2). Although fewer nests were discovered in 2013 than 2012, a far greater percentage of the primary nests discovered were active and thus destroyed while active in 2013 (83%) than 2012 (47%) (Table 1).

**Table 1:** Yearly totals of *Vespa velutina* nests

	2007	2008	2009	2010	2011	2012	2013	2014
Number of nests	4	27	83	61	77	94	90	111
Identified as primary nests	0	0	38	25	26	34	29	12
Identified as secondary nests	0	0	45	36	51	60	61	99
Active primary destroyed	–	–	18	4	11	16	24	0
Active secondary destroyed	–	–	14	18	22	34	39	79
Number of detected colonies	4	27	63	40	62	76	85	99

Most nests were destroyed after discovery, but only the destruction of active nests contributes to control of this pest. Fortunately, as this invasion progressed, a greater percentage of discovered nests (both primary and secondary) were destroyed while still active, from 2007 to 2014, respectively: 25, 44, 39, 36, 43, 53, 70 and 71% (Table 1). This suggests an increased awareness of *V. velutina*, and an increasingly active approach to locating nests earlier in the season. In 2014 a relatively low number of primary nests were discovered (Table 1) and none of these were discovered while active. A relatively large proportion of the secondary nests discovered in 2014 were discovered and thus destroyed while active.

There was a difference between number of nests discovered and number of nests destroyed in 2007, 2008 and 2009. In 2007, three nests were not destroyed; this is because they were found so late in the year, after the nest had ceased to be active. One nest in 2008 was recorded as not destroyed; however, this is due to blank data fields. Other nests recorded on the date were recorded as “partially destroyed by temperature”. As this was December, the nest would already have been inactive, so while the nest was not recorded as destroyed, it was correctly not included in the destroyed while active total. In 2014, it appears as though 11 nests were not destroyed, with the date of destruction being recorded as zero. There is no further evidence to suggest whether these nests were destroyed or not.

**Fig. 3:**
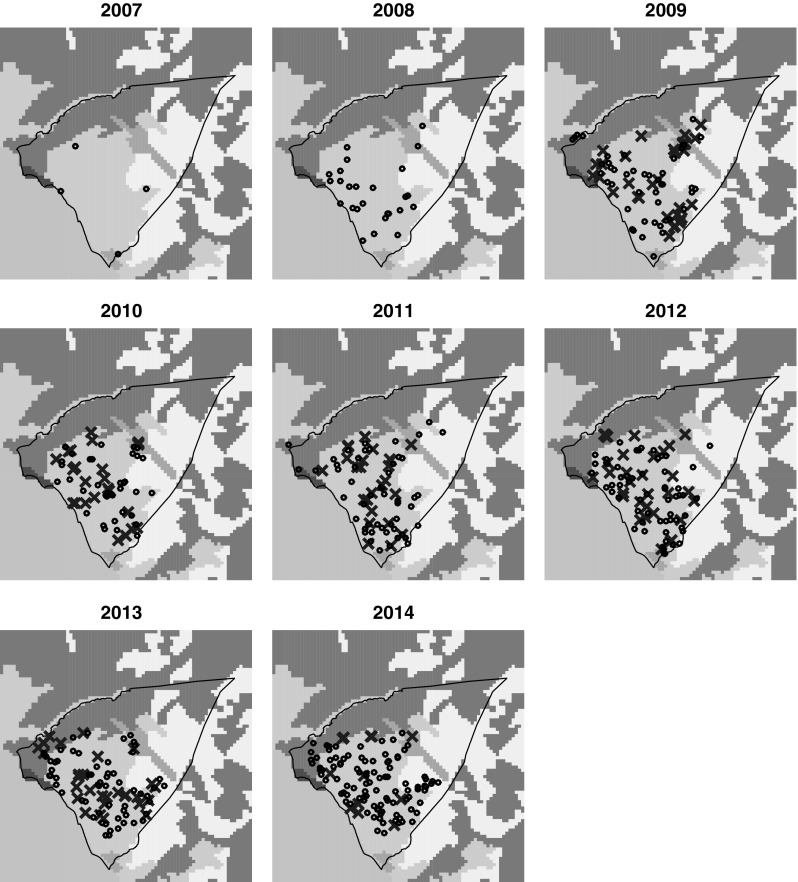
Nest locations over terrain of Andernos-les-Bains. Each map represents an 8 × 8 km square. Boundary of Andernos-les-Bains shown with *solid black line*. Terrain colours: *Light grey* continuous urban fabric; *dark grey* port; *pink* sports/leisure ground (region in the north identified as a grass airstrip); *green* coniferous forest; *pale green* broad leaved forest; *yellow* woodland shrub; *pale blue* sea. *Blue crosses* location of primary nests. *Black circles* locations of secondary nests. In 2007 and 2008, nests were not specified as primary or secondary (colour figure online)

The distribution and spread of *V. velutina* nests within Andernos-les-Bains from 2007 to 2014 and how this related to the terrain is shown in Fig. 3. Almost all reported nest locations were recorded as being in urban areas, which likely reflects both nesting preference and the ease of detection. Single nests were occasionally reported as being outside of the urban environment, but were generally close to the urban area, as opposed to the north-east of the commune which comprises mostly open countryside. The scale of the commune and the dispersal range of *V. velutina* make it impossible to infer which nest comes from which of the nests present in the previous year.

### Nest density

The density of nests fell slightly in 2013 suggesting that equilibrium was being reached, but the density markedly increased in 2014 (Table 2). The density figure is highly sensitive to a small change in population size, as the area considered is small.

**Table 2:** Density of *Vespa velutina* nests in Andernos-les-Bains

Year	2007	2008	2009	2010	2011	2012	2013	2014
Number of detected colonies	4	27	63	40	62	76	85	99
Density of nests across whole commune (nests per km^2^)	0.19	1.31	3.06	1.94	3.01	3.69	4.13	4.81
Density of nests within urban area in commune (nests per km^2^)	0.41	2.79	6.51	4.13	6.40	7.85	8.78	10.23

Last row represents the density of nests assuming all the nests in the commune were discovered in the urban area

### Nest habitats

**Fig. 4:**
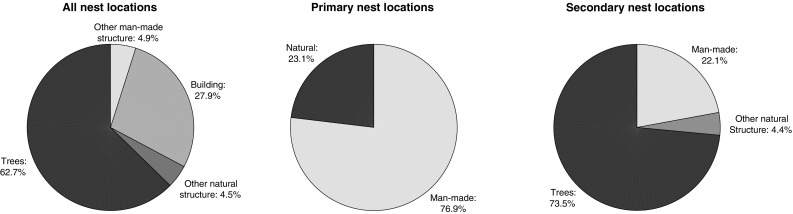
Structures that nests were built in

Of a combined 225 nests from the years 2007–2009 and 2014, 201 included the structure in which the nest was located, 135 (67.2%) of these were on a natural structure (Fig. 4), 62.7% of all nests were on trees (0.5% were in tree stumps), 27.9% of nests were in or on a building, 3 (1.5%) nests were reported as being on the underside of a manhole cover. The following list gives the tree types first as a percentage of the nests, and then as a percentage of total nests on trees.

Oak (*Quercus*), 81 (40.3%, 64.3%); pine (*Pinus*), 20 (10%, 15.9%); plane tree (*Platanus*), 5 (2.5%, 4%); poplar (*Populus*), 3 (1.5%, 2.4%); alder (*Alnus*), cedar (*Cedrus*) and locust tree (*Robinia*), 2 (1%, 1.6%); birch (*Betula*), tulip tree (*Liriodendron*), acacia (*Acacia*), lime tree (*Tilia*), sweetgum tree (*Liquidambar*) and fir (*Abies*), 1 (0.5%, 0.79%). In addition 4 (2%, 3.2%) nest structures were labelled just as tree.

An inexhaustive list of other structures includes: the bank of a brook, a ventilation grill, a road sign, a birdhouse, an electricity pylon, a magnolia bush/tree (not included in tree figures), a hedge and a bamboo plant.

The nest habitat differed between primary and secondary nests (Fig. 4). Of the 39 nests identified as primary and with a nest habitat, 30 (78.9%) were located in/on a man-made structure. Of the 136 nests identified as secondary nests and with a nest habitat, 106 (77.9%) were located in/on natural structures. One hundred of these were on trees.

### Parameter inference

The parameter inference echoed many of the findings already discussed from the more standard analyses of the data. The predicted total number of nests (discovered plus undiscovered) increased over the years, as did the ability to detect active nests. The two main surprises from this analysis are the relatively low between-year growth rate (*r*) of 9.64 [with 95% credible interval (CI) 8.41–10.94] and the relatively low proportion of nests that are detected. Concentrating on this latter effect, we predicted that 204 colonies (CI 124–339) were established in 2014, which contrasts with the 99 that were discovered. This difference may have two main contributing factors: firstly, there are likely regions of Andernos-les-Bains that are less thoroughly investigated, reducing the overall detection probability; secondly the Andernos-les-Bains region is not an isolated area and therefore the analyses will be including a number of nests beyond the administrative boundary that contribute to future nests.

In addition to providing more rigorous confidence intervals on all of the underlying mechanisms, this quantitative assessment can also be used to predict future dynamics. Without detection and destruction of nests the carrying capacity of colonies in the region was predicted to be 219 (CI 105–466), equating to a density of 10.64 (CI 5.10–22.63) nests per km^2^. However, assuming active detection levels remain at their maximum, this carrying capacity was reduced to around 166 (CI 86–307), which implied that in Andernos-les-Bains on average 15.6 (CI 11.5–20.4) active primary, 42.0 (CI 32.4–52.0) active secondary and 21.7 (CI 15.5–28.6) inactive secondary nests will be discovered annually. We therefore predict that even with the current high levels of detection only 51.4% (CI 29.5–79.0%) of nests are ever discovered and only 37.4% (CI 21.2–58.0%) of nests are discovered whilst active.

## Discussion

The data from Andernos-les-Bains reveal qualitative differences in the distribution of *V. velutina* to what was previously thought. The percentage of nests found underground (1.5%) was low, as the literature has previously suggested (Rortais et al. 2010). However, in Andernos-les-Bains a lower proportion of the nests were in trees compared to the previous study (Rortais et al. 2010), meaning there was a far higher percentage on or in buildings. The distribution of nests among trees also differed slightly from the Villemant report. Oak was again the most commonly nested in tree; however, nests in poplar trees were not common in our data, but this was the second most common tree species to be nested in as recorded in the Villemant report. Conifer trees (pine and cedar) showed a similar level of choice of nest habitat. The distribution of *V. velutina* nests is likely a consequence of the available locations, and particularly tree species, as the hornet is highly adaptable to differing environments. The distinction between primary and secondary nest habitats should be considered when searching for nests for the purposes of destruction. Primary nests are mostly found on or in man-made structures, typically buildings, and secondary nests are mostly located in trees. This order explains some of the difficulty in detection of *V. velutina* nests. The primary nests are in locations that are conducive to being sighted, but negating that, the nests are small. Secondary nests can be very large (Monceau et al. 2014b; Perrard et al. 2009), but are present in trees during the summer months when they will be obscured by foliage and likely far above eye level.

The calculation of the spatial densities of colonies within defined terrain groups is the first accurate figure produced to date, and an indication of the seemingly low level of competition between *V. velutina* colonies (Monceau and Thiéry 2016). The national record of nests in France was reported to have 1637 nests in a 160,000 km^2^ area (Rome et al. 2011), equating to a density of approximately 1 nest per 100 km^2^. This is clearly far smaller than the results from this more intensively sampled region. This difference is due to the nest reporting process, and represents large levels of under-reporting at the national scale; however, that data collection and its associated study serve a different purpose. The figures presented here may only represent a single example of *V. velutina* colonisation, but illustrate the high densities that can be reached despite human efforts at limiting its growth.

The parameter inference allows predictions to be made on the impact of nest destruction. Given the predicted carrying capacity of colonies was 166 with nest destruction, it can be said that the population size of *V. velutina* in Andernos-les-Bains will continue to grow. The estimation of carrying capacity without detection provides a useful figure from which to make predictions of total population size (and thus population density) in territories in which *V. velutina* are not currently present. The authors will be submitting for publication a simulation model of the potential invasion of *V. velutina* in England and Wales using the given density figures.

The observed population dynamics of *V. velutina* are not those of a natural system, and are strongly influenced by the effects of detecting and destroying nests. The notable decrease in the number of detected nests in 2010 is strong evidence that the effective destruction of many active nests in 2009 had an impact on the subsequent year. However, quantification of such interactions requires some form of mathematical model, which we match to the data using Bayesian methods. This analysis suggests that only around half of the nests that contribute to next-year’s nests are discovered, and that even with considerable effort placed into detection and destruction, *V. velutina* is likely to remain a major invasive pest.

Due to the method of data collection it is very likely that there was under-reporting of nests; this is supported by the detailed parameter inference. The records relied on nest sightings; the vigilance in detection likely increasing over time, as the knowledge and impact of *V. velutina* became better known. It also seems likely that those beekeepers whose hives were being preyed upon by *V. velutina* then searched for nests. The changes in proportions of nest types and nests found when active in 2014 suggests that the effort made in searching for *V. velutina* nests changed to be concentrated at a time of year when secondary nests were active, and a reduced effort was made in locating primary nests while active.

The location of almost all nests being within the urban area of the commune is likely a consequence of the data collection method. Accessibility and human density play a strong role in detection. Andernos-les-Bains does not have agricultural land and therefore cannot offer evidence for or against the report of a previous study in which almost as many nests were discovered in agricultural areas as urban areas (Villemant et al. 2011). The lack of this other preferable terrain may skew the density of nests towards the urban environment. The terrain around the urban area of the commune is mostly forest, which is 7 times less likely to have *V. velutina* nests (Villemant et al. 2011). Therefore, the low detection levels outside of the urban environment are likely a combination of true density patterns and detection effort bias. The carrying capacity calculated for Andernos-les-Bains may be more accurate as a carrying capacity for the urban terrain of Andernos-les-Bains. Assuming this to be the case, as 47% of the terrain in Andernos-les-Bains is urban, the carrying capacity of 219 equates to a density of 22.63 nests per km^2^.

The MCMC scheme does not consider the location of honeybees as a food source in the calculation of the carrying capacity. This is not considered critical, however, given the prey spectrum and opportunistic nature of *V. velutina* predation (Muller et al. 2013; Richter 2000; Villemant et al. 2011). The classification of whether a nest was active or inactive was necessary to make inferences on the impacts of nest destruction. Had the cut-off dates been later in the year, more nests would have been assumed destroyed whilst active, leading to a higher carrying capacity calculated from the MCMC scheme. While it is possible that a seemingly inactive secondary nest could have a hibernating queen inside, this only accounts for a small proportion of new queens that would have originated from that nest. Therefore, destruction of the nest would have little impact on the dynamics of the invasion spread.

The preliminary analysis of these data as a closed system is not ideal (Fig. 3), and this limitation is highlighted in the number of undiscovered secondary nests predicted to contribute to next year’s foundresses by the MCMC methodology. Andernos-les-Bains, while having sea to the south, has other communes and thus other urban areas to both the north-west and south-east. Given the expected dispersal distances of *V. velutina* are up to 30 km (Marris et al. 2011) and that these communes are also very likely to have *V. velutina* nests, the population dynamics of each of these areas may be interacting, potentially affecting the carrying capacity and time to reach that carrying capacity. Considering Andernos-les-Bains to be a closed system could lead to an overestimation of the carrying capacity. Measures to control and possibly eliminate *V. velutina* in Andernos-les-Bains would likely be more successful if neighbouring communities carried out similar and harmonised practices. Importantly, whilst *V. velutina* is a notifiable pest, actions taken against it are discretionary and therefore likely to be spatially heterogeneous.

The data and analysis presented here are a novel quantification of the dynamics of invading *V. velutina*. As such these findings provide the most informative basis for future investigations and potential models for the spread of *V. velutina* in other areas. The presented data represent a single realisation of the invasive dynamics of *V. velutina* and therefore cannot discriminate the effects of local climate and environment; however, we feel that this lack of variability is more than compensated for by the immense efforts to detect nests, providing a more reliable sample than could be obtained at a larger spatial scale. Moreover, the analyses suggest that the invasion of *V. velutina* is very difficult to control; the rapid spread of *V. velutina* between seasons, the high equilibrium densities and the difficulties of detecting nests in non-urban areas all contribute to this species’ overall success as an invasive pest. Given the recent incursion of *V. velutina* into Britain (National Bee Unit 2016), the knowledge of nest location preferences can directly aid efforts to halt the invasion, while the parameterisation, in particular the density of colonies, can be utilised directly in predictions of spread, providing vital information to Government and the beekeeping industry.
